# Plant Factory: A New Playground of Industrial Communication and Computing

**DOI:** 10.3390/s22010147

**Published:** 2021-12-27

**Authors:** Yu Liu, Sepehr Mousavi, Zhibo Pang, Zhongjun Ni, Magnus Karlsson, Shaofang Gong

**Affiliations:** 1 Department of Science and Technology, Campus Norrköping, Linköping University, 60221 Norrköping, Sweden; liuyu580@outlook.com (Y.L.); zhongjun.ni@liu.se (Z.N.); magnus.b.karlsson@liu.se (M.K.); 2 SweGreen AB, 11259 Stockholm, Sweden; sepehr.mousavi@swegreen.se; 3 ABB Corporate Research, 72226 Västerås, Sweden; pang.zhibo@se.abb.com

**Keywords:** plant factory, cyber-physical systems, Internet of Things, industrial IoT

## Abstract

Plant Factory is a newly emerging industry aiming at transforming crop production to an unprecedented model by leveraging industrial automation and informatics. However, today’s plant factory and vertical farming industry are still in a primitive phase, and existing industrial cyber-physical systems are not optimal for a plant factory due to diverse application requirements on communication, computing and artificial intelligence. In this paper, we review use cases and requirements for future plant factories, and then dedicate an architecture that incorporates the communication and computing domains to plant factories with a preliminary proof-of-concept, which has been validated by both academic and industrial practices. We also call for a holistic co-design methodology that crosses the boundaries of communication, computing and artificial intelligence disciplines to guarantee the completeness of solution design and to speed up engineering implementation of plant factories and other industries sharing the same demands.

## 1. Introduction to Plant Factory

### 1.1. Background

The term Plant Factory refers to a newly emerging form of agricultural production that caters to growing needs of crop production by human beings. With artificial control of the plant growing environment, a plant factory is expected to achieve high yield, cropping density, and economic return [1]. A similar concept is Vertical Farming [2] whereas Green Plant Wall [3] is another closely related product. All of them fall into the vision of Urban Agriculture [4] that envisions an agricultural industry integrated into an urban economic and ecological system.

As a rising industry fitting to the Industry 4.0 paradigm, there are massive opportunities to be expected in plant factories. Meanwhile, real-time monitoring of plant health, optimal control of the growing environment, and predictive and adaptive maintenance of the cropping system encounter grand challenges in terms of industrial communications [5] and flexible computing architectures [6] while industrial artificial intelligence (AI) [7] shall be considered as a driving force facing smart plant factories. However, till today, efforts for tackling these challenges are still isolated within individual disciplines.

In this paper, we first review the technical use cases, requirements and challenges of a plant factory, and then introduce recent progresses and state-of-the-art research of plant factories in the academic and industrial areas. As one of the major contributions, we dedicate an architecture that incorporates industrial communication and computing to the plant factory and provide a preliminary proof-of-concept to validate the feasibility, based on accumulated results in our academic and industrial practices. Future directions on development of plant factories are pointed out. We also call for a holistic co-design methodology that crosses the boundaries of communication, computing and AI disciplines to guarantee a good solution design and to speed up the engineering development in plant factories and other industries sharing the same demands.

### 1.2. Conceptual Architecture

**Figure 1:**
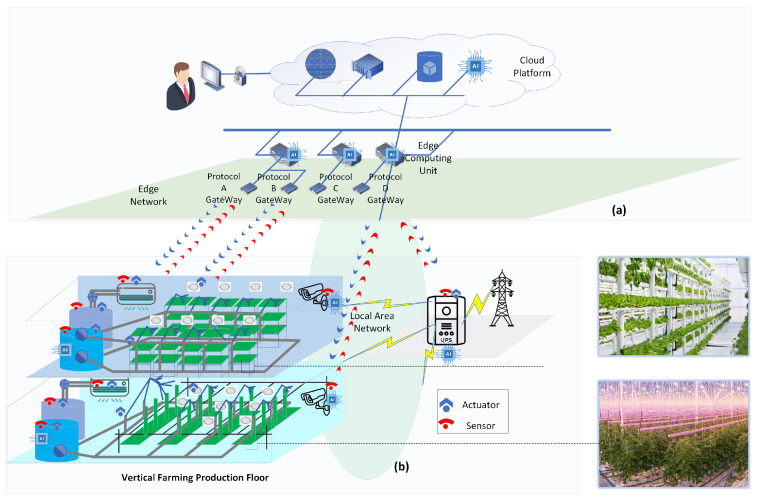
Conceptual architecture of the plant factory from (**a**) industrial informatics perspectives, (**b**) utilization of communication, computing, and AI.

Figure 1 depicts a conceptual architecture of a typical plant factory, which needs a synergy of industrial informatics (see Figure 1a) and operation technologies in the production floor where communication, computing and AI can play a role (see Figure 1b). In the crop production floor, plants are growing in vertically stacked modules. The stacked structure brings tremendous flexibility to the arrangement of different cultivars while providing standardized nursery services, leading to the maximum revenue. Crop productions in a plant factory highly rely on cooperation and interaction of a series of subsystems, such as illumination, air management, thermal control, nutrient delivery and factory automation systems, which covers the whole procedure from plant growth phases to harvest and transport. Benefiting from Internet of Things (IoT) in industry, massive sensors that give feedback on, e.g., temperature, relative humidity, CO_2_, pH value and electric conductivity, are deployed in the plant factory to have a comprehensive understanding of the growing environment to provide a precise control of plant growing status. A camera-based monitoring system is also exploited for plant health monitoring. Sensory data are collected through different wired and wireless communication protocols, accumulated and analyzed in edge computing units and in the cloud in a real-time manner to continuously optimize the plant growth strategy with AI. Meanwhile, the operation status of automation facilities is also monitored so that predictive maintenance can be scheduled. Moreover, the emergence of plant factories also brings broad application opportunities to other disciplines, such as power electronics, mechanics, control systems, and robotics, but the scope of this article concentrates on the communication and computing domains.

### 1.3. Use Cases, Requirements, and Challenges

The success of a plant factory highly relies on three subsystems, namely the light-emitting diode (LED) supplemental system, the plant growth system, and the environmental control system, which appeal for different requirements on industrial communication, computing as well as intelligence. A typical plant factory can be 400 times more efficient in land use compared to outdoor farming [8], thanks to the vertically structured growing modules. As a result, massive sensors that monitor indoor climate and automation facilities, and actuators for the growth system are widely distributed in a plant factory, indicating that wireless communications are preferred to ease the deployment. Communications among massive sensors and actuators are usually with low data rates and low data volume, but the high density of node distribution can bring significant challenges, especially to the co-existence of multiple protocols operating on shared frequency bands. Moreover, an appropriate localization for a specific device is also demanded for plant care or maintenance purposes. Meanwhile, real-time monitoring of plant health demands high-speed and high-resolution image/video transmission services, requiring broadband network support. Apart from these latency-tolerable services, remote operations on automation facilities and robotics, require ultra-low latency and deterministic real-time performance. In addition to local area networks, the logistics for food supply chain needs a wide area network to provide low-cost positioning and product tracking services.

The modular design of a plant factory implies that a proper distribution of the computing units is required, as the computation cannot be completely carried out on the field production floor or loaded to the cloud. For example, the modular design enables the flexibility to cultivate crops of different types and different growing phases in the same space, which requires that the LED supplemental lighting shall be highly customizable to provide differentiated spectrum and intensity to accommodate each cultivar, so do other modules, such as the nutrient delivery, air management and thermal control systems. On the one hand, the computing of a supervisory controller must be performed within individual modules with the intent of improving the system scalability and reducing the latency of control to guarantee the quality of service (QoS). On the other hand, plant factories aim for a fully controlled environment agriculture (CEA), to which a closed-loop production principle is applied, indicating that all processes of advanced crop cultivation shall be operated in an optimized manner [2]. This is where cloud and edge computing can play a significant role. In a typical plant factory, millions of sensor data points are collected every day [8]. Data analytics in huge amounts must be handled in the cloud to guarantee the control strategy of subsystems is dynamically evolving with time while edge computing can effectively accelerate the real-time data processing and enable fast decision-making to the production field. Consequently, an appropriate computing architecture and partitioning of tasks to different computing units lead to the maximum efficiency of a plant factory.

Recent advancements in machine learning and deep learning have witnessed the landing of AI in industrial processes. As for plant factories, deep involvement of industrial AI is inevitable, seeing the tremendous potentials in terms of automating the optimization of plant growth systems, and plant health monitoring and diagnosis, which needs further concrete research.

## 2. State of the Art

A series of leading companies within the plant factory and vertical farming market are surveyed and some of the representatives are listed in Table 1, ranging from farming companies to system integrators. Current landscape of farming companies is formed by early vertical farming companies that continuously evolve with time (e.g., Spread), and new startups that are backed by modern industrial automation and informatics (e.g., Iron Ox), but none has shown dominating power in the market. With specialized expertise, system integrators mostly dedicate themselves to a niche market, i.e., the market of LED supplemental lighting, plant growth system, or control system.

Today’s plant factory and vertical farming industry are still in a primitive phase, especially from a technological perspective. First, even though a fully autonomous plant factory for either aeroponics or hydroponics is on the blueprint of the industry, the automation in general is quite limited. Sowing, harvesting, and even growing module management are highly relying on human labor. The low degree in automation constraints that current farming companies can only produce a few variations of lettuce due to a lack of food processing ability. Second, communications are mainly based on wired solutions, which brings huge burdens to installation and maintenance, let alone the complex cabling and aging effect that can result in long term headaches. Third, most computing tasks are placed in the production field while cloud computing is only used for data storage, presentation, and remote human interactions. This is partially due to the lack of high degree automation and the absence of intelligence. A majority of the companies apply a pre-set strategy-based or grower-assisted control strategy while the introduction of AI has only been witnessed in offline data analytics by professionals. Therefore, the feedback loop and iterations are far behind the requirement for real-time analytics and autonomous control in a closed loop that are envisioned by the industry, although many (e.g., Plenty ag and Techno Farm) have claimed AI will be a pillar in their future plant factories.

In the academic world, discussions about plant factory and vertical farming are usually under the concept of smart agriculture. In [9,10], general technologies in each discipline, such as sensing, communication, cloud computing and automation that can contribute to smart agriculture, are reviewed while vertical farming is not specifically focused. Researches explicitly discussing vertical farming are more from a social impact perspective. The authors in [11,12] have a quantified and qualified evaluation of energy demand and carbon footprint of vertical farming, emphasizing its significance to city sustainability and climate change. Although the technology development for vertical farming is much more radical than the industry within a single discipline, such as LED lighting [13], software control system [14], machine learning [15] and big data [16], these single-disciplinary innovations are far from an integrated and systematic solution that is demanded by the industry.

In short, a modernized plant factory requires harmonization of industrial communication, computing and AI, which has not been witnessed in today’s plant factory practice and research. Besides, there are still several common obstacles constraining the employment of industrial IoT in plant factories, e.g., interoperability and compatibility among different communication standards, device management of heterogeneous hardware, reliable security scheme. In addition, data analytics on real-time and historical data with multiple data sources, rates, formats, and storage locations pose significant challenges to the current design paradigm of industrial automation networks. These requirements and challenges can hardly be resolved with isolated efforts put into individual disciplines. Therefore, a new design paradigm is under demand catering to the practical needs of the plant factory.

## 3. Preliminary Proofs-of-Concept

**Figure 2:**
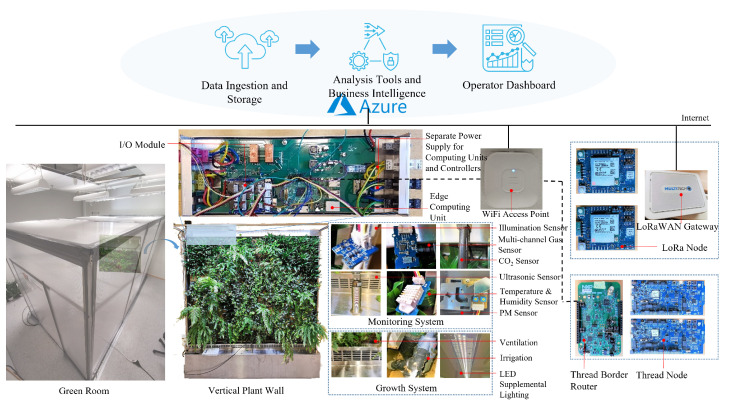
A preliminary study of the architecture of a plant factory: field infrastructure.

In light of the aforementioned requirements of the plant factory, in this section, we propose a preliminary architecture as a start point for vertical farming, which is based on our previous accumulated research results and digital transformation practice in the vertical plant wall industry [17,18,19,20,21]. Figure 2 depicts the infrastructures in an indoor green room where a vertical plant wall is deployed with monitoring and growth systems, which reflects the production field of a plant factory. Environmental sensors that continuously collect the temperature, humidity, illumination intensities of ultraviolet, infrared and visible light, CO_2_, particulate matter (PM) and volatile organic compound (VOC) parameters are installed to the plant wall to provide real-time monitoring of the indoor climate. A high-precision ultrasonic sensor is utilized to detect water level changes so as to enable fine-grained monitoring and control of water consumption. Moreover, actuators, such as a stepless pulse-width modulation (PWM)-controlled fan, a water pump, and two-channel LED strips, are deployed as ventilation, irrigation and supplemental lighting systems to guarantee the growth of the plants as well as adjust the indoor climate. The sensors and actuators are connected to an edge computing unit using different wired or wireless communication protocols. The edge computing unit is deployed as a bridge between the field devices, i.e., sensors and controllers, and the cloud. It aggregates device-to-cloud telemetries from sensors and receives cloud-to-device commands so as to update local control strategies for actuators accordingly. Furthermore, the edge computing unit can also operate in an offline mode, which to a large extent guarantees a stable growth of plants. On the edge computing unit, a microcontroller component processes input/output (I/O) operations that require deterministic timing and a microprocessor component is responsible for bidirectional communications between local devices and the remote cloud platform.

The proposed framework incorporates three distinguishing communication protocols, namely WiFi, Thread and LoRaWAN, to provide wireless communication support. The Thread protocol, which is designed for building automation purposes, can seamlessly integrate sensors and actuators into existing building automation networks. The native support of Internet Protocol (IP) in WiFi and Thread enables massive innovations in the Internet world to be brought into the realm of industrial IoT while LoRaWAN enables extremely low-power and wide-area communication for low complexity sensors. In our proof implementation, Thread-based sensors can communicate with the edge computing unit through a Thread border router device, which relays messages between a Thread mesh network and the Ethernet so as to enable bidirectional communication. LoRa-based devices can periodically update telemetries to the cloud through a LoRaWAN gateway. With this approach, the proposed framework can cover both wired and wireless communications, and features both local area and wide-area network scenarios that are demanded by plant factories.

**Figure 3:**
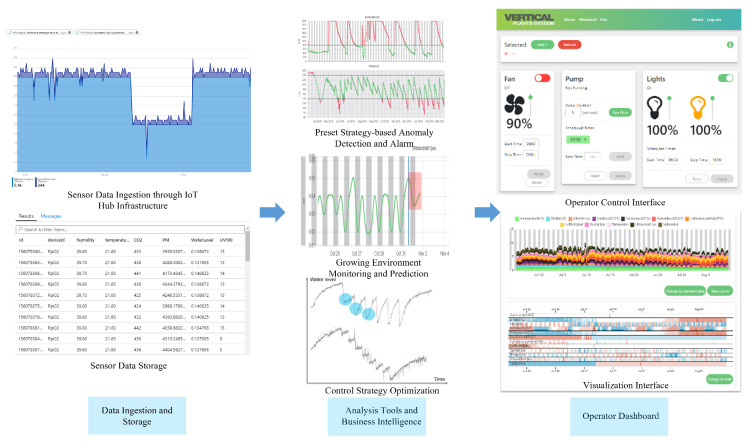
A preliminary study of the architecture of a plant factory: cloud infrastructure.

The cloud-based computing services are exemplified with the Azure cloud infrastructure, as shown in Figure 3. In the cloud, three segments of services are implemented, namely data ingestion and storage, analysis tools and business intelligence, and an operator dashboard. An IoT Hub service is employed as a device registry and a message broker. Based on the IoT Hub infrastructure, our solution features a unified digital twin-based device management model and a broker-based data model. For each industrial IoT device deployed in the plant factories, two identical digital twin objects of the device are created at the device and in the cloud upon device registration. By proactive synchronization of the digital twins, the device can be remotely monitored and manipulated from the cloud. Meanwhile, messages between field devices deployed in the plant factories and the other cloud services are also routed through IoT Hub to eliminate the interoperability gap between devices, and to enhance the security with a consistent security scheme. Implementation details about the communication and computing framework are presented in [17,18]. With collected data from field devices and sufficient computing resources in the cloud, threshold-based alarm functions and neural network-based anomaly detection for indoor climate are implemented to optimize the control strategy of the plant growth system. For instance, the energy consumption of irrigation is largely reduced by analyzing the water level in the tank. Furthermore, an auto-encoder (AE) and long short-term memory encoder-decoder (LSTM-ED) based neural network models are developed to detect point anomalies and contextual anomalies in the indoor environment, respectively, which exhibits the integration of AI into the system [21]. An intuitive dashboard is developed for operators to have an insight of the production field and to interact with the system.

Seeing the trend that container-based virtualization and orchestration technologies have become a cornerstone of edge computing, in our implementation, several computing tasks are containerized and migrated from the cloud to the edge to ensure large-scale deployment and to guarantee a fast response to critical tasks in the production field such as controlling the water pump. Meanwhile, machine learning tasks such as anomaly detection of indoor climate can also be deployed as microservices and flexibly upgraded in an online approach, which are detailed in [19]. In general, this preliminary study showcases an early implementation for future plant factories, which covers communication, computing as well as AI use cases.

An increased automation level in the plant factory will lead to more mission-critical tasks and demand high-performance communications, which cannot be achieved with the aforementioned framework. The 5G technology is expected to become key supporting assets for industrial communications with superior performance in high data rate and low latency [5]. In addition to that, in [22,23,24,25], the Six-port technology is researched. With simplified modulation and demodulation using direct baseband data conversion, it is targeting at ultra-low latency (<1 ms) and high-speed (>10 Gbps) communication in the industry. In [26], a completely customized protocol stack, WirelessHP, is proposed for high performance critical industrial control applications, which is able to reach multi-Gbps data rate, sub-microsecond scheduling unit and very high reliability. The research in [27,28] show low-loss metamaterial for ultra-high-speed data transmission at low cost. These studies have the potential to disrupt the landscape of current industrial communication technologies, thus may benefit future communications in plant factories, though delay time from software layer running on edge computing units and in the cloud needs further investigation.

**Figure 4:**
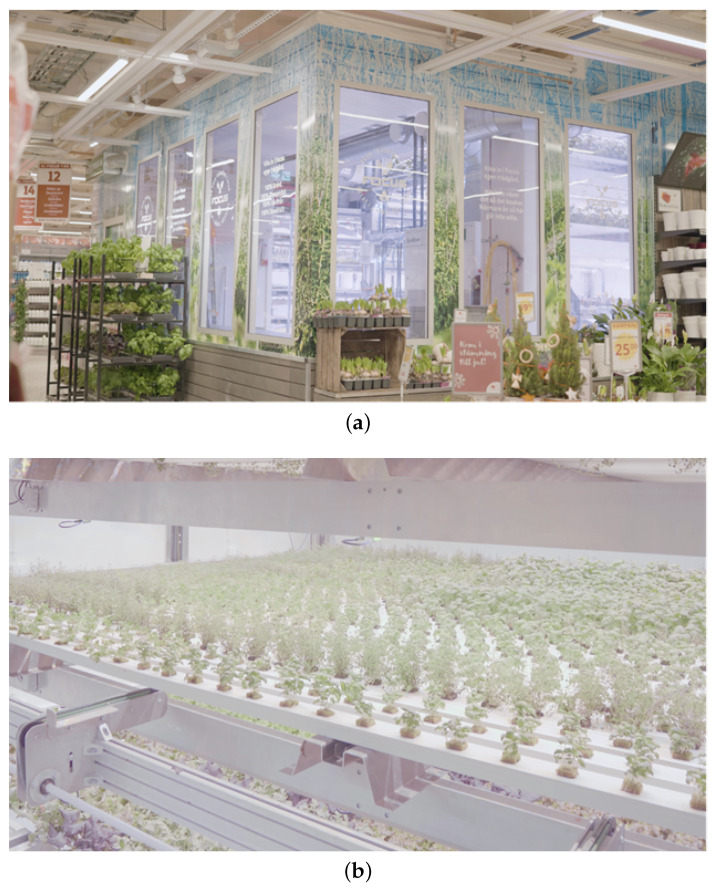
(**a**) SweGreen’s industrial practice of vertical farming in a store in Sweden. (**b**) Growing tracks of a fully-automated farming as a service unit of SweGreen in the testbed environment [29].

The vision for the plant factory described above has also been preliminarily put into practice and verified by the vertical farming industry. The Swedish agriculture company SweGreen is a promising example. SweGreen developed a new business model, namely farming as a service (FaaS), to provide in-store solutions for hyper-local production of leafy vegetables [29]. Figure 4a,b depict a plant factory testbed implemented by SweGreen which is deployed in a local store in Linköping, Sweden. In the testbed, a cloud-based sensing system and advanced automation technologies are utilized to achieve two purposes of ‘Unit Management’ and ‘Farm Management’. For the unit management, the key is to guarantee optimal parameters for the growing of vegetables through real-time monitoring and precise control. These parameters can be divided into two categories. The first is indoor climate, which typically includes temperature, relative humidity, airflow, and light intensity level. The other is nutrient elements, which include the three main elements, namely nitrogen, phosphorus, and potassium, and over 17 minor elements such as manganese and magnesium. The aforementioned parameters can add up to more than 150 kinds, all of which require real-time monitoring and precise control. For the farm management, AI-based planning tools are used to schedule the farming actions and provide farm operators with daily action guidelines to match the customer/retailer’s harvest schedule. Through such a FaaS, the full life cycle of plants production is within a 100% controlled environment and the so-called business intelligence (BI) is implemented. As a further step, a holistic approach is needed to provide more precise control and better planning, and ultimately to achieve autonomous management of both unit and farm.

## 4. A Holistic Methodology

During the solution design procedure, we have realized that a comprehensive consideration of the entire automation network was inevitably necessary, especially from an engineering implementation perspective. Therefore, we call for a holistic methodology that crosses the boundaries of industrial communication, computing and AI disciplines, to tackle the challenges encountered by the plant factory. A new solution design shall consider potential contributions from all the three disciplines. This brings two major advantages, namely completeness and high efficiency. In the former case, for instance, to guarantee the QoS of critical applications such as power management or automation systems, the ultra-low latency in radio communications and the determinism of the software layer running on computing units shall be achieved in parallel. Otherwise, the improvement in one layer is likely to be mitigated by the latency introduced in another layer, leading to the deterioration of general system performance. Similarly, the security requirement shall be satisfied in both communication and computing, covering all the components in the system.

By breaking the boundaries, the challenge in one discipline can be tackled by innovations from other disciplines, which greatly improves the flexibility and efficiency in deploying new solutions. For example, with AI-capable hardware deployed to the edge computing units, AI algorithms can partially or entirely be applied to the edge so that the intelligence gets involved in production processes in an earlier manner. In this context, appropriate partitioning of tasks into edge units and the cloud server shall be investigated and scheduled. Furthermore, powered by AI, dynamic resource allocation and context caching in edge devices contribute to the lift of network performance and co-existence of protocols in the communication layer. In this way, the holistic co-design methodology envisions a deep integration of industrial communication, computing and intelligence to unleash the power of innovation in all disciplines and elevate the feasibility of solution design, to satisfy the diverse application requirements posed by the plant factory.

## 5. Conclusions and Future Directions

The vision of Industry 4.0 foresees that many traditional industries will be reshaped by the evolution of information technologies, represented by IoT and cloud computing. The upcoming Industry 5.0 vision is a further step to that enhanced by edge computing, cloud-based automation and AI technologies. In light of this trend, a concept of plant factory is proposed as an anticipated step facing the digital revolution in the agricultural industry, which aims at transforming the crop production to an unprecedented model by leveraging factory automation and industrial informatics.

Today’s plant factory and vertical farming are at the very beginning phase to accumulate experiences and verify technologies, and far from the picture envisioned by Industry 4.0 & 5.0. Considering densely deployed sensors in a plant factory, using wireless communication and cellular networks [30] to eliminate cables becomes an overwhelming choice for the future. The low degree of automation leaves considerable space for traditional industrial and process automation players to contribute. Moreover, industrial AI shall play a fundamental role in indoor climate monitoring, plant growth control and optimization, as well as plant health monitoring and diagnosis. AI will also facilitate plant health monitoring, to reach closed loop and autonomous control in real-time. The participation of automation and AI would exponentially increase the computing load, which demands an evolution in computing architecture. Therefore, a properly partitioning of cloud computing, edge computing and field computing needs to be investigated.

However, current industrial cyber-physical systems are not fully pledged for the solution design of a plant factory in terms of communication, computing and AI. We present our preliminary studies to exhibit how industrial communications, edge/cloud computing and AI can be integrated to a unified framework for the plant factory, to tackle some of the challenges, such as interoperability among different protocols, high-performance communications, and smart manufacturing and maintenance. The vision has also been validated by a preliminary deployment of an urban farming testbed by the real industry. We also call for a holistic co-design methodology that crosses the boundaries of the three tightly related disciplines. A comprehensive consideration of communication, computing, and AI in an industrial automation network is the key to elevating system performance. This methodology can be a guideline for further solution design and engineering practices.

In addition to upstream production, a mature plant factory and vertical farming industry also look forward to innovations in middle stream and downstream, i.e., the food processing and food supply chain. We started research on food supply chain many years ago [31]. An IoT-enabled systematic solution was proposed and consolidated with prototyping, aiming at transforming the food supply chain with added values. Today, with evolving communication technologies, powerful computing capability, and rapidly evolving AI, the food supply chain deserves more upgrades to guarantee quality control and maximize business value.

In summary, this article reviews the state of the art of the plant factory, points out the future requirements of a modern plant factory and the deficiency in today’s industry, while presenting a preliminary system design. It can be referenced by other industries experiencing similar needs. However, considering the fact that the development of plant factories is still at a primitive phase, more studies on deeper cooperation of industrial communication, computing and AI must be conducted to achieve a practical plant factory with both IoT and AI.

## Figures and Tables

**Table 1:** Leading farming companies and system integrators in the plant factory and vertical farming industry.

Category	Company	Solution Name	Systems			Communication	Computing	Artificial Intelligence	Control Strategy
			LED Supplemental Lighting	Growth System	Control System				
Farming Company	Plenty ag	None				Not disclosed	Field	Claim being used	Grower- assisted control
	AeroFarms	None				Not disclosed	Cloud	Predictive analytics of growing environment	Grower-assisted remote monitoring and control
	Iron Ox	None				Wired	Field	Not disclosed	(Targeted at) high level autonomous control
System Integrator	Heliospectra	ELIXIA helioCORE				Wireless (WiFi) Wired(Ethernet)	Field and cloud	None	Pre-set strategy-based control
	Smart Grow	FRAMEZ				Wired	Field	None	Manual control
	Lumigrow	TopLight BarLight				Wireless	Field	None	Pre-set strategy-based control
	Illumitex	FarmVisionAI				Wired	Cloud	Image analytics of plants	Manual control
	Crop One	Crop One Farm Manager				Wired	Field and cloud	Data analytics for cultivar-specific growth support	Grower-assisted monitoring and control
	Certhon	Certhon Phytotron Control System				Wired	Field and cloud	Not disclosed	Grower-assisted monitoring and control
	Priva	Priva Compact CC				Wired(Ethernet)	Field and cloud	None	Pre-set strategy-based control

## Data Availability

Data sharing not applicable.
